# 

**Published:** 1890-12-06

**Authors:** 


made beef-tea, &c., whereas had JOHNSTON'S BOVRIL been used in their stead, the patients would in nine cases out of ten have gained strength to battle against the disease under which they were suffering. I have no hesitation to advise you [illegible]scribe to your patients, to your convalescents, to those of your clients who have mental exertions, to use JOHNSTON'S [illegible], which, in a concentrated form, contains a substantial tonic and a palatable food."—J. M. BEAUSOLIEL, M.D.

[illegible] THROUGHOUT THE UNITED KINGDOM.

### THE HOSPITAL

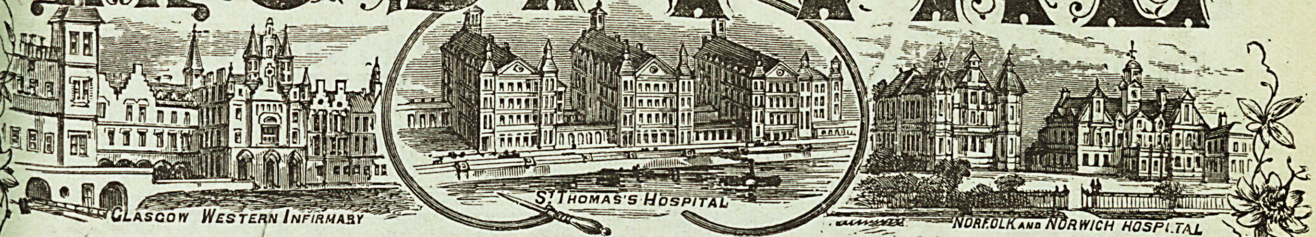

WITH EXTRA NURSING SUPPLEMENT.

No. 219. Vol. IX.] Saturday, December 6th, 1890. [One Penny.

